# Supra-Acetabular Brown Tumor due to Primary Hyperparathyroidism Associated with Chronic Renal Failure

**DOI:** 10.1100/tsw.2010.86

**Published:** 2010-05-04

**Authors:** Rosaria M. Ruggeri, Emanuele Calamoneri, Antonia Russo, Alessandro Sindoni, Baldassarre Mondello, Maurizio Monaco, Michele A. Rosa, Sergio Baldari, Salvatore Benvenga, Alfredo Campennì, Francesco Trimarchi

**Affiliations:** ^1^ Department of Clinical-Experimental Medicine and Pharmacology, Division of Endocrinology, University of Messina, Messina, Italy; ^2^ Department of Surgical Sciences, University of Messina, Messina, Italy; ^3^ Department of Radiological Sciences, Division of Nuclear Medicine, University of Messina, Messina, Italy; ^4^ Division of Cardiovascular and Thoracic Sciences, University of Messina School of Medicine, Messina, Italy

**Keywords:** bone scintigraphy, chronic renal disease, brown tumor, normocalcemia, primary hyperparathyroidism

## Abstract

A 63-year-old woman presented to the Orthopedic Unit of our hospital complaining of right hip pain of 6 months'duration associated with a worsening limp. Her past medical history included chronic renal insufficiency. Physical examination revealed deep pain in the iliac region and severe restriction of the right hip's articular function in the maximum degrees of range of motion. X-rays and CT scan detected an osteolytic and expansive lesion of the right supra-acetabular region with structural reabsorption of the right iliac wing. ^99m^Tc-MDP whole-body bone scan showed an abnormal uptake in the right iliac region. Bone biopsy revealed an osteolytic lesion with multinucleated giant cells, indicating a brown tumor. Serum intact PTH was elevated (1020 pg/ml; normal values, 12 62 pg/ml), but her serum calcium was normal (total = 9.4 mg/dl, nv 8.5–10.5; ionized = 5.0 mg/dl, nv 4.2–5.4) due to the coexistence of chronic renal failure. ^99m^Tc-MIBI scintigraphy revealed a single focus of sestamibi accumulation in the left retrosternal location, which turned out to be an intrathoracic parathyroid adenoma at surgical exploration. After surgical removal of the parathyroid adenoma, PTH levels decreased to 212 pg/ml. Three months after parathyroidectomy, the imaging studies showed complete recovery of the osteolytic lesion, thus avoiding any orthopedic surgery. This case is noteworthy because (1) primary hyperparathyroidism was not suspected due to the normocalcemia, likely attributable to the coexistence of chronic renal failure; and (2) it was associated with a brown tumor of unusual location (right supra-acetabular region).

## INTRODUCTION

Primary hyperparathyroidism (PHPT) is a relatively frequent condition that predominantly affects elderly women and is usually associated with asymptomatic, mild to moderate, chronic hypercalcemia. The prevalence of PHPT is estimated at $3/10^3$ in the general population. It is three times more common in women than men and the average age at diagnosis is 55 years[1,2].

Morbidity from PHPT includes skeletal deterioration, nephrolithiasis and nephrocalcinosis, gastrointestinal symptoms, as well as nonspecific symptoms of fatigue and mood alterations. Symptoms are often subtle and may be stable for many years[3]. Most cases are asymptomatic for a long time or even lifelong[4].

Diffuse bone demineralization is common. Patients with long-standing and moderately severe disease may exhibit characteristic bone changes, including periosteal reabsorption of the distal phalanges, salt and pepper appearance of the skull, and tapering of the distal clavicle. Benign brown tumors and bone cysts, although relatively rare, may also occur and may be mistaken for primary skeletal neoplasms[3,5]. The early detection and treatment of PHPT led to a marked decrease in the appearance of the said classical bone and renal manifestations that characterize the chronic disease. Surgical resection of the benign adenoma is generally curative[4,5,6].

We herein describe the uncommon case of a supra-acetabular brown tumor due to a mediastinal hyperfunctioning parathyroid adenoma manifesting with normocalcemia, likely attributable to the coexistence of chronic renal failure.

## CASE REPORT

A 63-year-old woman was referred to the Orthopedic Unit of our hospital complaining of right hip pain of almost 6 months' duration associated with a worsening limp. Due to the progressive bone pain, the patient was not able to either stand or walk. Her past medical history included degenerative joint disease, osteoporosis, hypertension treated with angiotensin receptor antagonists and alpha-blockers at standard doses, and chronic renal insufficiency of 5 years' duration, which was managed conservatively with hypoproteic diet. Clinical examination revealed deep pain related to the iliac region and severe restriction of the right hip's articular function in the maximum degrees of range of motion.

Imaging studies (X-rays and CT scan) detected an osteolytic and expansive lesion of the right supra-acetabular region with structural reabsorption of the right iliac wing (Fig. 1). Whole-body bone scintigraphy with $^{99m}$Technetium methylene-diphosphonate ($^{99m}$Tc-MDP) displayed an abnormal uptake in the right supra-acetabular region, as well as a number of areas of increased activity in some ribs (fifth and ninth right ribs, and fourth and seventh left ribs), left sternoclavicular joint, right iliac wing, and long bones (distal right femur, and upper and mid tibiae) (Fig. 2). Percutaneous biopsy of the right iliac bone revealed an osteolytic lesion replaced by proliferating fibroblastic cells, spindle cells, and multinucleated giant cells, consistent with the diagnosis of brown tumor.

The patient was referred to the Endocrinology Unit for further investigation. Serum intact PTH was measured using a solid-phase, two-site, chemiluminescent, enzyme-labeled, immunometric assay (Immulite 2000) and was markedly elevated (1020 pg/ml; normal values 12–62 pg/ml in our laboratory). However, due to the coexisting renal failure, serum calcium level was normal (total calcemia = 9.4 mg/dl; nv 8.5–10.5; ionized calcemia = 5.0 mg/dl, nv 4.2–5.4). Serum phosphorus was low-normal (2.7 mg/dl; nv 2.5–4.5 mg/dl). The 24-h urinary calcium and phosphorus were decreased (56 mg/24 h with nv of 100–300 and 394 mg/24 h with nv of 400–1000, respectively). Due to kidney failure, blood urea nitrogen (106 mg/dl) and creatinine (3 mg/dl) were elevated, whereas creatinine clearance was decreased (28.6 mg/24 h). PHPT was suspected because of the very high levels of PTH (never checked before by her nephrologists in the presence of normal values of serum calcium and phosphorus) and because of the few years' duration of the renal failure. Neck ultrasonography (US) showed a large intrathoracic multinodular goiter. No parathyroid enlargement was clearly detectable at US, likely due to the conspicuous enlargement

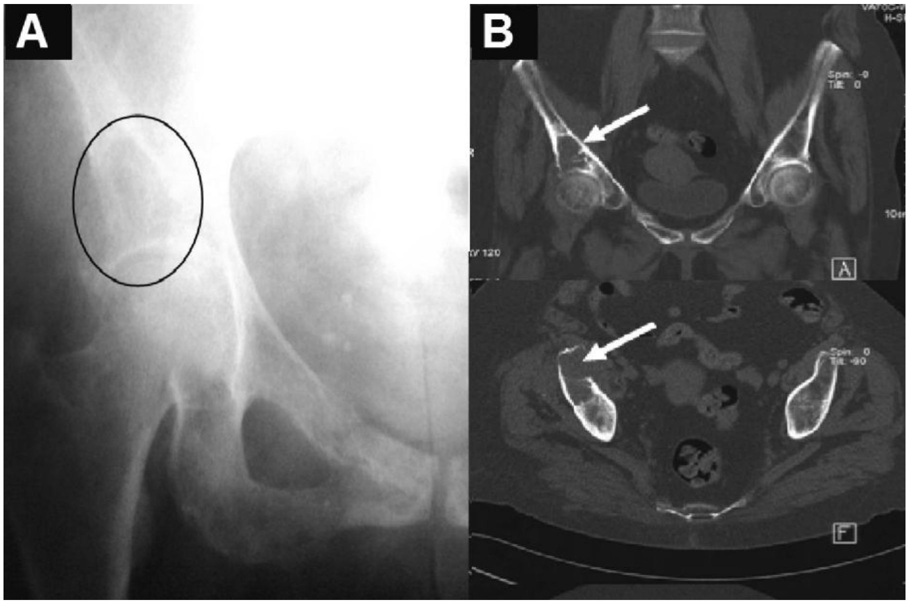

**FIGURE 1**. (A) X-rays detected an expansive osteolytic lesion of the right supra-acetabular region. (B) CT scan confirmed an expansive osteolytic lesion of the right supra-acetabular region with structural reabsorption of the right iliac wing.

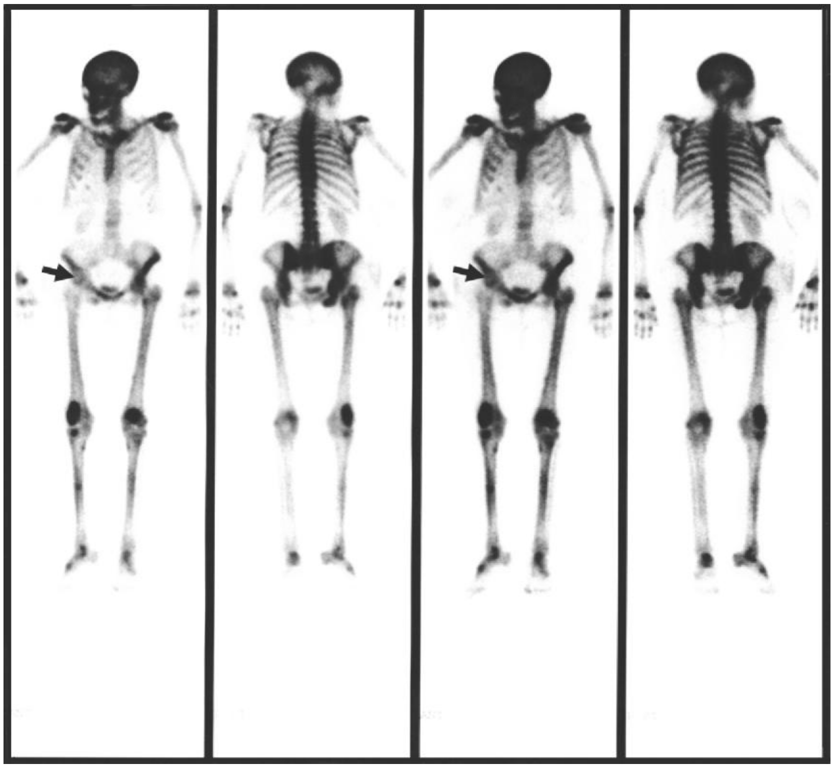

**FIGURE 2**. Whole-body bone scintigraphy with $^{99m}$Tc-MDP, showing an irregular ring of increased uptake of the tracer in the right supra-acetabular region (black arrow), as well as areas of increased activity in the fifth and ninth right ribs, fourth and seventh left ribs, left sternoclavicular joint, right iliac wing, distal right femur, and upper and mid tibiae.

and multinodularity of the thyroid. Parathyroid planar scintigraphy using dual-phase $^{99m}$Tc-methoxyisobutyl isonitrile (MIBI) revealed a single focus of sestamibi accumulation in the left retrosternal location (Fig. 3). This finding was consistent with a parathyroid adenoma, which was ectopically located in the left mediastinum. CT scan of the neck and thorax confirmed the presence of a mediastinal mass of 3 cm in diameter positioned close to the left brachiocephalic vein. The patient underwent surgery at our Thoracic Surgery Unit. At exploration, an enlarged left parathyroid adenoma measuring 3 × 2 cm was removed, along with the multinodular goiter (subtotal thyroidectomy). Pathological examination of the surgical specimens was consistent with a benign parathyroid adenoma and a colloid goiter, respectively. After surgery, PTH levels decreased to 212 pg/ml, thus indicating parathyroid gland sparing.

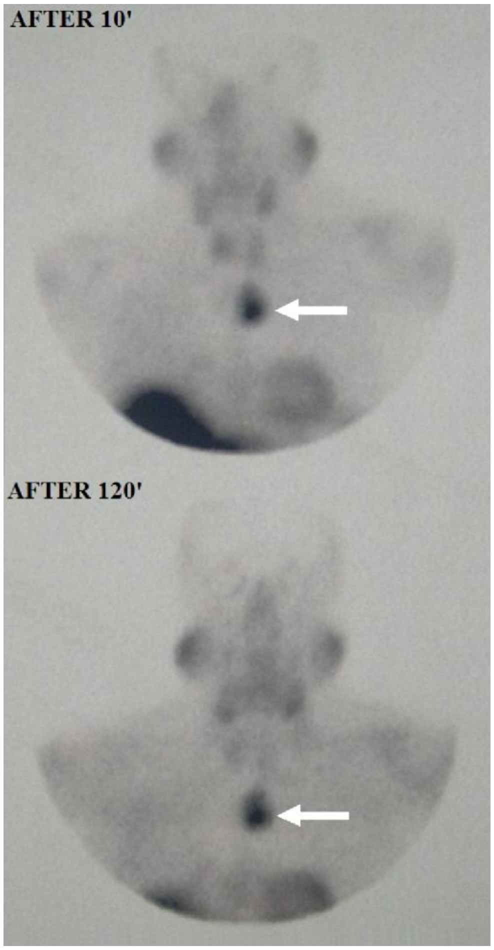

**FIGURE 3**. Parathyroid scintigraphy ($^{99m}$Tc-MIBI) demonstrated pathological and intense uptake of the tracer in a definite lesion localized just under the jugulum (anterosuperior mediastinum) (white arrow).

Three months after surgery, the patient’s bone pain progressively resolved, so that she could walk again. The imaging studies showed a substantial regression of the supra-acetabular lesion, as well as the regression of the other osteolytic lesions detected at bone scintigraphy (Fig. 4). Thus, there was no need to perform the scheduled surgical curettage and bone cement stabilization of the lesion in order to decrease both patient pain and the fracture risk.

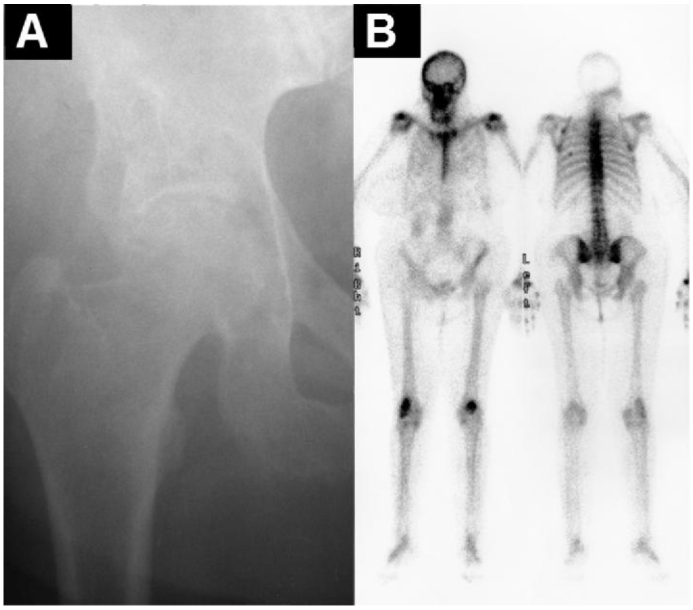

**FIGURE 4**. (A) X-ray showing a substantial regression of the supra-acetabular lesion 3 months after surgical removal of the parathyroid adenoma. (B) Whole-body bone scintigraphy with $^{99m}$Tc-MDP, showing the substantial regression of the supra-acetabular lesion, as well as of the other osteolytic lesions previously detected, 3 months after surgery.

## DISCUSSION

Herein, we have described the clinical presentation, management, and outcome of a case of severe PHPT manifesting with a brown tumor of unusual localization.

Histologically, the classical "brown tumor" of hyperparathyroidism is a collection of osteoclasts mixed with innocuous spindle cells and poorly mineralized woven bone[5,7]. It may mimic giant-cell tumors of bone, which may be confused with malignancies in the absence of appropriate clinical and imaging information. Even though the "brown tumor" can localize anywhere in the skeleton, the preferential locations are the head bones (particularly the mandible), and the ends of long bones and ribs[8,9,10,11,12,13,14]. Obviously, an unusual brown tumor localization may render the differential diagnosis of skeletal osteolytic lesions difficult. Occurrence of a brown tumor in the supra-acetabular region, as in our patient, appears to be quite rare. To the best of our knowledge, only three cases of presentation of this abnormality have been cited in the literature[13,14,15].

The first-line treatment of brown tumors involves the correction of hyperparathyroidism, which usually leads to tumor regression[5,7]. Orthopedic surgery should be considered if the mass does not regress after parathyroidectomy and progress with spontaneous fracture risk, or if bone pain is still present[13]. In our patient, the lesion decreased in size and recalcified after parathyroidectomy, thus avoiding any additional orthopedic procedure.

Although brown tumors are one of the most pathognomonic signs of hyperparathyroidism, they have become extremely rare clinical entities due to the relatively early diagnosis of PHPT because of the routine assay of serum calcium[3,5]. In our patient, PHPT was overlooked – and the diagnosis delayed – because serum calcium levels were within the normal range. Normocalcemia was the result of two divergent changes: hypercalcemia from the hyperfunctioning parathyroid adenoma and hypocalcemia from the coexisting chronic renal insufficiency.

The phenomenon of the normocalcemic PHPT has been debated in the literature for many years, with data on the incidence and natural history of this phenotype being limited[4]. Scanty data also exist on the presence of brown tumors in normocalcemic PHPT[16,17,18]. Recent studies have addressed the clinical relevance of this condition[19] and have demonstrated that such patients have more substantial skeletal involvement than in typical hypercalcemic PHPT and develop more evident signs and complications over time[19]. In the absence of secondary causes of hyperparathyroidism, it has been proposed that these normocalcemic patients may represent the most precocious expression of PHPT[19]. Nevertheless, several disorders, e.g., vitamin D deficiency[16] or asymptomatic celiac disease[20], should be considered as possible underlying causes in some cases of normocalcemic PHPT.

In our patient, the PHPT was masked by the chronic renal disease, a well-known cause of secondary hyperparathyroidism (SHPT)[21]. The vitamin D deficiency, due to the decreased capacity of the kidney to synthesize 1,25$(OH)_2$ D in renal failure, and the consequent impairment of calcium absorption in the intestine, as well as the decreased excretion of phosphate-lowering calcium levels, could account for the normal calcemia in this patient. It is well known that PHPT with coexisting vitamin D deficiency may present with normocalcemia[22]. Moreover, in patients with chronic renal insufficiency, parathyroid autonomy can develop because of genetic changes that result in clonal expansion of cells, leading to autonomous function of one or more glands. Finally, in this patient, the few years' duration of the chronic renal failure makes improbable the development of a SHPT with severe skeletal lesions as a brown tumor.

## CONCLUSION

We have reported the uncommon case of a supra-acetabular brown tumor due to a mediastinal hyperfunctioning parathyroid adenoma, which illustrates the importance of considering chronic renal failure as a possible underlying cause of normocalcemic PHPT (and not of SHPT only). Moreover, even though serum calcium levels are routinely checked by the majority of clinicians, PTH levels should also be checked for differential diagnosis, as this case confirms, in cases of expanding osteolytic bone lesions.
